# *N*-(2-Bromo­phen­yl)thio­urea

**DOI:** 10.1107/S1600536810008305

**Published:** 2010-03-10

**Authors:** Halima F. Saleem, Bohari M. Yamin

**Affiliations:** a School of Chemical Sciences and Food Technology, Universiti Kebangsaan Malaysia, UKM 43600 Bangi Selangor, Malaysia

## Abstract

In the title compound, C_7_H_7_BrN_2_S, the thio­urea unit is almost perpendicular to the bromo­benzene fragment, making a dihedral angle of 80.82 (16)°. The crystal structure is stabilized by N—H⋯S inter­molecular hydrogen bonds, which form linear chains along the *ab* diagonal.

## Related literature

For bond-length data, see: Allen *et al.* (1987 ▶). For related structures, see: Steiner (1998 ▶); Shen & Xu (2004 ▶); Wang *et al.* (1991 ▶). For the anti­viral activity of phenyl­thio­ureas, see: D’Cruz & Uckun (2005 ▶); Frank & Smith (1955 ▶); Mao *et al.* (2000 ▶); Sudbeck *et al.* (1998 ▶).
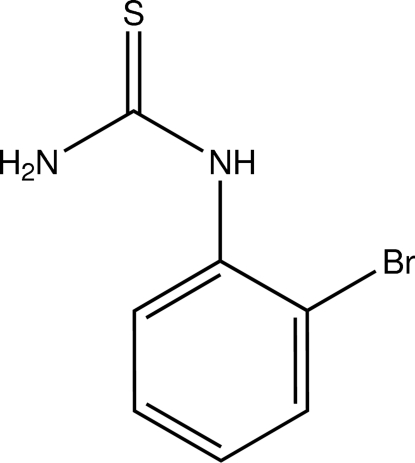


## Experimental

### 

#### Crystal data

C_7_H_7_BrN_2_S
*M* *_r_* = 231.12
Monoclinic, 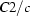

*a* = 15.181 (3) Å
*b* = 7.7952 (16) Å
*c* = 15.312 (3) Å
β = 90.803 (4)°
*V* = 1811.8 (6) Å^3^
*Z* = 8
Mo *K*α radiation
μ = 4.71 mm^−1^
*T* = 298 K
0.44 × 0.27 × 0.11 mm

#### Data collection

Bruker SMART APEX CCD area-detector diffractometer
Absorption correction: multi-scan (*SADABS*; Bruker, 2000 ▶) *T*_min_ = 0.231, *T*_max_ = 0.625
5817 measured reflections
1972 independent reflections
1327 reflections with *I* > 2σ(*I*)
*R*_int_ = 0.028

#### Refinement

*R*[*F* ^2^ > 2σ(*F* ^2^)] = 0.046
*wR*(*F* ^2^) = 0.129
*S* = 1.06
1972 reflections
100 parameters
H-atom parameters constrained
Δρ_max_ = 0.61 e Å^−3^
Δρ_min_ = −0.65 e Å^−3^

### 

Data collection: *SMART* (Bruker, 2000 ▶); cell refinement: *SAINT* (Bruker, 2000 ▶); data reduction: *SAINT*; program(s) used to solve structure: *SHELXS97* (Sheldrick, 2008 ▶); program(s) used to refine structure: *SHELXL97* (Sheldrick, 2008 ▶); molecular graphics: *ORTEP-32* for Windows (Farrugia, 1997 ▶) and *PLATON* (Spek, 2009 ▶); software used to prepare material for publication: *SHELXTL* (Sheldrick, 2008 ▶), *PARST* (Nardelli, 1995 ▶) and *PLATON*.

## Supplementary Material

Crystal structure: contains datablocks global, I. DOI: 10.1107/S1600536810008305/dn2543sup1.cif

Structure factors: contains datablocks I. DOI: 10.1107/S1600536810008305/dn2543Isup2.hkl

Additional supplementary materials:  crystallographic information; 3D view; checkCIF report

## Figures and Tables

**Table 1:** Hydrogen-bond geometry (Å, °)

*D*—H⋯*A*	*D*—H	H⋯*A*	*D*⋯*A*	*D*—H⋯*A*
N1—H1*A*⋯S1^i^	0.86	2.54	3.354 (3)	161
N2—H2*A*⋯S1^ii^	0.85	2.53	3.368 (3)	168

Symmetry codes: (i) 
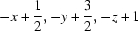
; (ii) 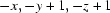.

**Fig. 1.**
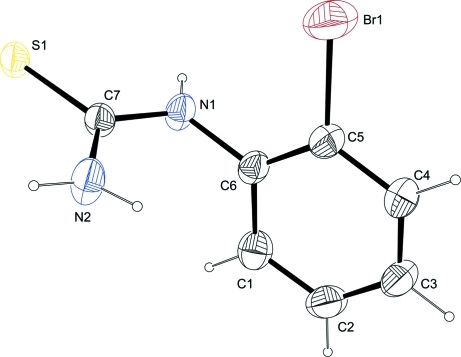
The nolecular structure of (I), with the atom labeling scheme. Displacement ellipsods are drawn at the 30% probability level. H atoms are represented as small spheres of arbitrary radii.

**Fig. 2.**
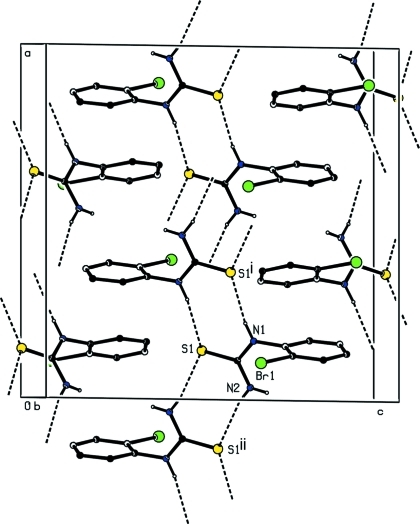
A packing diagram of (I) viewed down the b axis. Hydrogen bonds are shown by dashed lines. Hydrogen atoms not involved in hydrogen bondings have been omitted for clarity. [Symmetry codes: (i) -x+1/2, -y+3/2, -z+1; (ii) -x, -y+1, -z+1.]

## supplementary crystallographic information

### Comment

The number of publications including patents on the application of thiourea compounds in the field of pharmaceutical is increasing at a considerable rate. The antivarial activities of a series of phenylthioureas as none-nucleoside inhibitors HIV-1 reverse transcriptase (NNRTIs) with efficacy against multi-drug resistant viruses (Sudbeck *et al.*, 1998; Mao *et al.*, 2000; D'Cruz & Uckun, 2005) are some of the interesting examples. Several *N*-thiourea compounds of the type H_2_NC(S)NHR are now commercially available.

The title compound (I) is analagous to phenylthiourea (II, Shen *et al.*, 2004), *o*-fluorophenylthiourea (III, Steiner, 1998) and *p*-bromophenylthiourea(IV, Wang *et al.*, 1991). The thiourea moiety, S1/N1/N2/C7, and the 2-bromoaniline fragment, Br1/N1/(C1—C6) are each planar with maximum deviation of 0.024 (5)Å for C2 atom from the least square plane. The two planes are perpendicular to each other with dihedral angle of 80.82 (16)° compare to 68.57° in (IV). The thiourea moiety maintains its cis-trans geometry. The bond lengths and angles are in normal ranges (Allen *et al.*, 1987) and comparable to those in (II), (III) and (IV). In contrast to its fluoro- analog ,the molecule is stablized only by pairs of N1—H1A···S1 and N2—H2A···S1 (symmetry codes as in Table 1) intermolecular hydrogen bonds to form linear chains along the diagnal of the ab face (Fig.2).

### Experimental

The compound was prepared by the method described by Frank & Smith (1955) with a slight modification. Ammonium thiocyante (0.38 g, 0.005 mol) in 15 ml acetone was added into 20 ml acetone solution of containing benzoylchloride (0.70 g, 0.005 mole). The solution was filtered and the filtrate was kept into a 100 ml two neck round bottom flask. *o*-Bromoaniline (0.86 g, 0.005 mole) was added into the flask and the mixture was refluxed for 2 hours. The final solution was poured into a baker containing some ice cubes. The precipitate formed was filtered. The precipitate was then added into a beaker containing 50 ml aqueous solution of sodium hydroxide (7 g). The solution was heated to boiling for 10 minutes. After a week on standing at room temperature some colourless crystals were obtained and found suitable for X-ray investigation. The yield was 81% and melting point; 428.1-429.3 K.

### Refinement

H atoms on the C atoms were positioned geometrically with C—H= 0.93 for aromatic group and constrained to ride on their parent atoms with U_iso_(H)= 1.2 x U_eq_(C parent atom). The hydrogen atoms attached to the nitrogen atoms were located from the Fourier map and initially refined with U_iso_(H)= 1.2 x U_eq_(N) . In the last stage of refinement, they were treated as riding on their parent N atoms.

### Crystal data

**Table:**

C_7_H_7_BrN_2_S	*F*(000) = 912
*M**_r_* = 231.12	*D*_x_ = 1.695 Mg m^−^^3^
Monoclinic, *C*2/*c*	Mo *K*α radiation, λ = 0.71073 Å
Hall symbol: -C 2yc	Cell parameters from 1400 reflections
*a* = 15.181 (3) Å	θ = 2.6–27.0°
*b* = 7.7952 (16) Å	µ = 4.71 mm^−^^1^
*c* = 15.312 (3) Å	*T* = 298 K
β = 90.803 (4)°	Block, colourless
*V* = 1811.8 (6) Å^3^	0.44 × 0.27 × 0.11 mm
*Z* = 8	

### Data collection

**Table:**

Bruker SMART APEX CCD area-detector diffractometer	1972 independent reflections
Radiation source: fine-focus sealed tube	1327 reflections with *I* > 2σ(*I*)
graphite	*R*_int_ = 0.028
Detector resolution: 83.66 pixels mm^-1^	θ_max_ = 27.0°, θ_min_ = 2.6°
ω scan	*h* = −19→19
Absorption correction: multi-scan (*SADABS*; Bruker, 2000)	*k* = −5→9
*T*_min_ = 0.231, *T*_max_ = 0.625	*l* = −19→19
5817 measured reflections	

### Refinement

**Table:**

Refinement on *F*^2^	Primary atom site location: structure-invariant direct methods
Least-squares matrix: full	Secondary atom site location: difference Fourier map
*R*[*F*^2^ > 2σ(*F*^2^)] = 0.046	Hydrogen site location: inferred from neighbouring sites
*wR*(*F*^2^) = 0.129	H-atom parameters constrained
*S* = 1.06	*w* = 1/[σ^2^(*F*_o_^2^) + (0.0664*P*)^2^ + 1.1624*P*] where *P* = (*F*_o_^2^ + 2*F*_c_^2^)/3
1972 reflections	(Δ/σ)_max_ < 0.001
100 parameters	Δρ_max_ = 0.61 e Å^−^^3^
0 restraints	Δρ_min_ = −0.65 e Å^−^^3^

### Special details

**Table:**

Geometry. All esds (except the esd in the dihedral angle between two l.s. planes) are estimated using the full covariance matrix. The cell esds are taken into account individually in the estimation of esds in distances, angles and torsion angles; correlations between esds in cell parameters are only used when they are defined by crystal symmetry. An approximate (isotropic) treatment of cell esds is used for estimating esds involving l.s. planes.
Refinement. Refinement of *F*^2^ against ALL reflections. The weighted *R*-factor wR and goodness of fit *S* are based on *F*^2^, conventional *R*-factors *R* are based on *F*, with *F* set to zero for negative *F*^2^. The threshold expression of *F*^2^ > σ(*F*^2^) is used only for calculating *R*-factors(gt) etc. and is not relevant to the choice of reflections for refinement. *R*-factors based on *F*^2^ are statistically about twice as large as those based on *F*, and *R*- factors based on ALL data will be even larger.

### Fractional atomic coordinates and isotropic or equivalent isotropic displacement parameters (Å^2^)

**Table:**

	*x*	*y*	*z*	*U*_iso_*/*U*_eq_	
Br1	0.10527 (4)	1.16677 (8)	0.59724 (3)	0.0909 (3)	
S1	0.13874 (6)	0.57559 (13)	0.47185 (6)	0.0488 (3)	
N1	0.16731 (19)	0.7869 (4)	0.60361 (19)	0.0498 (8)	
H1A	0.2173	0.7961	0.5790	0.060*	
N2	0.0356 (2)	0.6495 (5)	0.6038 (2)	0.0660 (11)	
H2A	−0.0030	0.5879	0.5780	0.079*	
H2B	0.0218	0.7159	0.6460	0.079*	
C1	0.1703 (3)	0.7627 (6)	0.7636 (3)	0.0565 (10)	
H1	0.1868	0.6482	0.7587	0.068*	
C2	0.1620 (3)	0.8362 (6)	0.8439 (3)	0.0648 (12)	
H2	0.1754	0.7731	0.8939	0.078*	
C3	0.1338 (3)	1.0031 (7)	0.8512 (3)	0.0661 (12)	
H3	0.1267	1.0510	0.9063	0.079*	
C4	0.1163 (3)	1.0993 (6)	0.7788 (3)	0.0645 (11)	
H4	0.0972	1.2122	0.7845	0.077*	
C5	0.1267 (2)	1.0289 (5)	0.6969 (2)	0.0511 (9)	
C6	0.1536 (2)	0.8622 (5)	0.6873 (2)	0.0447 (9)	
C7	0.1116 (2)	0.6777 (4)	0.5653 (2)	0.0422 (8)	

### Atomic displacement parameters (Å^2^)

**Table:**

	*U*^11^	*U*^22^	*U*^33^	*U*^12^	*U*^13^	*U*^23^
Br1	0.1262 (6)	0.0874 (4)	0.0595 (3)	0.0326 (3)	0.0145 (3)	0.0182 (3)
S1	0.0499 (5)	0.0498 (5)	0.0472 (5)	−0.0151 (4)	0.0139 (4)	−0.0146 (4)
N1	0.0425 (17)	0.0608 (19)	0.0465 (17)	−0.0151 (15)	0.0159 (14)	−0.0186 (15)
N2	0.0445 (18)	0.087 (3)	0.067 (2)	−0.0264 (18)	0.0218 (16)	−0.036 (2)
C1	0.052 (2)	0.057 (2)	0.060 (2)	0.004 (2)	0.0012 (19)	−0.008 (2)
C2	0.072 (3)	0.077 (3)	0.045 (2)	−0.010 (2)	0.005 (2)	0.007 (2)
C3	0.080 (3)	0.076 (3)	0.042 (2)	−0.014 (3)	0.011 (2)	−0.013 (2)
C4	0.084 (3)	0.056 (2)	0.054 (2)	0.001 (2)	0.016 (2)	−0.014 (2)
C5	0.056 (2)	0.055 (2)	0.0418 (19)	−0.0019 (19)	0.0074 (17)	−0.0036 (17)
C6	0.0402 (19)	0.054 (2)	0.0403 (19)	−0.0100 (17)	0.0091 (15)	−0.0098 (16)
C7	0.0391 (19)	0.044 (2)	0.0440 (18)	−0.0072 (15)	0.0077 (15)	−0.0072 (15)

### Geometric parameters (Å, °)

**Table:**

Br1—C5	1.891 (4)	C1—C6	1.421 (6)
S1—C7	1.693 (4)	C1—H1	0.9300
N1—C7	1.331 (4)	C2—C3	1.375 (7)
N1—C6	1.428 (4)	C2—H2	0.9300
N1—H1A	0.8551	C3—C4	1.361 (6)
N2—C7	1.320 (4)	C3—H3	0.9300
N2—H2A	0.8506	C4—C5	1.380 (5)
N2—H2B	0.8562	C4—H4	0.9300
C1—C2	1.364 (6)	C5—C6	1.371 (5)
			
C7—N1—C6	124.0 (3)	C2—C3—H3	119.6
C7—N1—H1A	115.0	C3—C4—C5	119.8 (4)
C6—N1—H1A	120.1	C3—C4—H4	120.1
C7—N2—H2A	119.1	C5—C4—H4	120.1
C7—N2—H2B	117.4	C6—C5—C4	120.8 (4)
H2A—N2—H2B	121.2	C6—C5—Br1	120.0 (3)
C2—C1—C6	119.6 (4)	C4—C5—Br1	119.1 (3)
C2—C1—H1	120.2	C5—C6—C1	118.6 (3)
C6—C1—H1	120.2	C5—C6—N1	122.2 (4)
C1—C2—C3	120.2 (4)	C1—C6—N1	119.1 (3)
C1—C2—H2	119.9	N2—C7—N1	117.6 (3)
C3—C2—H2	119.9	N2—C7—S1	121.6 (3)
C4—C3—C2	120.9 (4)	N1—C7—S1	120.8 (3)
C4—C3—H3	119.6		
			
C6—C1—C2—C3	2.9 (6)	Br1—C5—C6—N1	0.6 (5)
C1—C2—C3—C4	−1.9 (7)	C2—C1—C6—C5	−2.1 (6)
C2—C3—C4—C5	0.0 (7)	C2—C1—C6—N1	176.5 (4)
C3—C4—C5—C6	0.8 (6)	C7—N1—C6—C5	−103.7 (4)
C3—C4—C5—Br1	−178.2 (3)	C7—N1—C6—C1	77.7 (5)
C4—C5—C6—C1	0.2 (6)	C6—N1—C7—N2	6.2 (6)
Br1—C5—C6—C1	179.2 (3)	C6—N1—C7—S1	−172.3 (3)
C4—C5—C6—N1	−178.3 (4)		

### Hydrogen-bond geometry (Å, °)

**Table:**

*D*—H···*A*	*D*—H	H···*A*	*D*···*A*	*D*—H···*A*
N1—H1A···S1^i^	0.86	2.54	3.354 (3)	161
N2—H2A···S1^ii^	0.85	2.53	3.368 (3)	168

Symmetry codes: (i) −*x*+1/2, −*y*+3/2, −*z*+1; (ii) −*x*, −*y*+1, −*z*+1.
